# A Top-Performing Algorithm for the DREAM3 Gene Expression Prediction Challenge

**DOI:** 10.1371/journal.pone.0008944

**Published:** 2010-02-04

**Authors:** Jianhua Ruan

**Affiliations:** Department of Computer Science, University of Texas at San Antonio, San Antonio, Texas, United States of America; Center for Genomic Regulation, Spain

## Abstract

A wealth of computational methods has been developed to address problems in systems biology, such as modeling gene expression. However, to objectively evaluate and compare such methods is notoriously difficult. The DREAM (Dialogue on Reverse Engineering Assessments and Methods) project is a community-wide effort to assess the relative strengths and weaknesses of different computational methods for a set of core problems in systems biology. This article presents a top-performing algorithm for one of the challenge problems in the third annual DREAM (DREAM3), namely the gene expression prediction challenge. In this challenge, participants are asked to predict the expression levels of a small set of genes in a yeast deletion strain, given the expression levels of all other genes in the same strain and complete gene expression data for several other yeast strains. I propose a simple 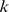-nearest-neighbor (KNN) method to solve this problem. Despite its simplicity, this method works well for this challenge, sharing the “top performer” honor with a much more sophisticated method. I also describe several alternative, simple strategies, including a modified KNN algorithm that further improves the performance of the standard KNN method. The success of these methods suggests that complex methods attempting to integrate multiple data sets do not necessarily lead to better performance than simple yet robust methods. Furthermore, none of these top-performing methods, including the one by a different team, are based on gene regulatory networks, which seems to suggest that accurately modeling gene expression using gene regulatory networks is unfortunately still a difficult task.

## Introduction

One of the fundamental goals in computational systems biology is to model gene expression levels, and to use such models to predict the behavior of the cell under various external/internal conditions. In recent years, a plethora of algorithms have been developed towards this goal (for example, see reviews [1]–[7]). A critical issue, however, is that such algorithms are often hard to be objectively evaluated or compared [8]. DREAM, which stands for Dialogue on Reverse Engineering Assessments and Methods, is an annual international event aimed at providing an unbiased platform to evaluate the strengths and weaknesses of computational methods in systems biology [8]. Each year, DREAM organizers provide a set of challenge problems in systems biology, e.g. to reverse-engineer gene regulatory networks or signaling networks, and invite scientists to solve them by computational approaches. The true solutions to the problems are held unknown to the participants at the time of prediction, which makes the evaluation relatively objective and unbiased [8], [9].

This paper presents a winning algorithm for one of the challenge problems, the gene expression prediction problem, in the third DREAM (DREAM3) event. For this challenge problem, participants are given gene expression time course data for four different strains of *S. Cerevisiae* - one wild type and three deletion strains - treated with some chemical. Participants are asked to predict the *relative* expression of a small subset of genes (prediction targets) in one of the deletion strains (prediction strain), given complete expression data for all four strains except the expression data for the prediction targets in the prediction strain. In addition, the identities of all genes are disclosed, and participants are free to use any publicly available data, such as gene expression data under other conditions, whole-genome ChIP-chip data, and functional annotations.

Predicting gene expression itself is of relatively low interest in practice, as biologists can easily measure gene expression with experimental methods such as DNA microarray or quantitative RT-PCR. The value of this challenge, therefore, lies in finding out whether gene expression can be accurately predicted, and what models can do the best job in predicting gene expression. Answers to these two questions are fundamentally important in many gene expression-based studies. Furthermore, many methods have been proposed for constructing gene regulatory networks [10]–[17], which are bases for modeling gene expression. Results of this challenge problem may tell us whether the current gene regulatory network models are sufficiently accurate to make quantitative predictions.

Popular gene regulatory network models include Bayesian networks [10]–[12], Boolean networks [13], and regression/classification-based models [14]–[17]. These methods can model the expression level of a gene by the expression levels of other genes [10], [13], [15], [16], by the presence or absence of TF binding sites on its promoter sequences [12], [14], [17], or a combination of the two types of information [18]–[20]. For this particular challenge problem, these methods can all potentially be applied, as most of them have been developed based on yeast data, and participants are allowed to use additional data beyond what was provided by the DREAM organizers.

Hypothesizing that the current regulatory network model may not be accurate enough to make quantitative predictions (see Discussion), I opted to use a different strategy, based on gene co-expression networks. The intuition is that if two genes are co-expressed in the wild type and two deletion strains, they might also be co-expressed in the third deletion strain, given that the deleted genes in the three deletion strains are known to be involved in similar biological processes. Therefore, I construct a co-expression network using a 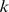-nearest-neighbor (KNN) method, where each gene is connected to 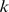 other genes with whom its expression profile is most similar. The expression of a prediction target under a prediction condition is then estimated to be the average of the expression levels of its 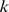 nearest neighbors, under the same condition. Interestingly, this idea coincides with one of the simplest missing data imputation methods [21]. Indeed, the challenge problem is exactly an example of a missing value estimation problem, for which many algorithms have been developed [21].

This simple method turns out to work well. Among the nine methods that made the final predictions, it shares the “best performer” honor with a much sophisticated method, which is based on soft integration of multiple data types using elastic net [22]. The performance of the two top-ranked algorithms is almost identical, and is much better than that of the other participating methods. In addition, I also proposed several alternative strategies, all based on simple ideas for missing value imputation. These results were not submitted to the challenge organizers officially (but were developed without knowing the ground truth). In particular, a modified KNN method achieved even better accuracy than the standard KNN method. Another KNN-based approach did not improve over the standard KNN, while a regression-based approach had slightly lower accuracy than the KNN-based methods. These results, together with the fact that none of the top-performing methods are trying to explicitly construct gene regulatory networks seem to confirm my hypothesis that current gene regulatory models are probably not accurate enough to model gene expression yet. In addition, the results also suggest that simple methods should in general be preferred over complex ones.

The remainder of this paper is organized as follows. In the next section, I first present the challenge problem, and then describe the prediction results I submitted to DREAM3. I also present the results from several alternative strategies and discuss the difference between several evaluation methods for measuring prediction accuracy. I then discuss some lessons learned from my participation in this challenge. In the last section I describe some details of the prediction methods and the evaluation methods.

## Results

### The Gene Expression Challenge Problem

In this gene expression prediction challenge, participants were given gene expression time course data for four different strains of *S. Cerevisiae*: wild type (wt), GAT1 deletion strain (gat1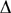), GCN4 deletion strain (gcn4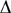), and LEU3 deletion strain (leu3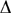). GAT1, GCN4, and LEU3 are all yeast transcription factors, and have functions in regulating nitrogen or amino acid metabolism genes [23]. Gene expression levels were assayed in each strain from eight time points (t = 0, 10, 20, 30, 45, 60, 90 and 120 minutes) following the addition of 3-aminotriazole (3AT), which is an inhibitor of an enzyme in the histidine biosynthesis pathway. Time t = 0 means the absence of 3AT. Microarray experiments were conducted using Affymetrix yeast genome array, with two biological replicates per sample. The data were normalized using the RMA algorithm [24] in the GeneSpring software. Data were provided by Neil Clarke from Genome Institute of Singapore.

The challenge is to predict the relative expression of a set of 50 selected genes in the gat1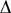 strain, given the complete expression data for all four strains, except the expression data to be predicted. The identities of all genes are disclosed, and participants are free to use any publicly available data. According to the challenge specification, absolute expression levels are neither required nor desired. It is recommended that the 50 genes should be ranked according to their relative induction or repression relative to the expression levels observed in the wild-type parental strain in the absence of 3AT, such that the gene with the highest induction has a rank 1 and the gene with the highest repression has a rank 50.

### Prediction Using 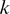-Nearest Neighbors

My final submitted prediction results are based on the simplest 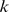-nearest neighbor (KNN) model. In this model, the expression level of a gene is predicted by taking the average of its 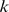 nearest genes. Similarities are measured according to Euclidean distances. To select a 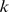 for the best model, a set of randomly selected genes is used to estimate the prediction accuracy of each model (see Methods for model selection and evaluation).

**Figure 1:**
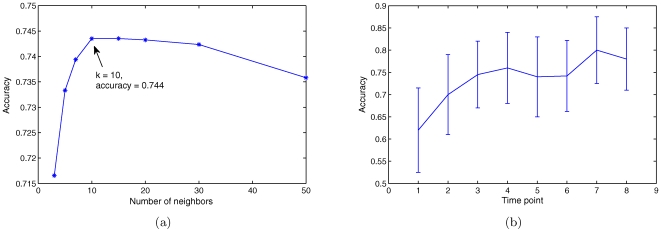
Prediction accuracy on randomly chosen genes. (a) Gene-profile accuracy as a function of 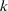. (b) Gene-profile accuracy for each time point.

Figure 1(a) shows the overall gene-profile accuracy of the KNN model on randomly chosen genes, as a function of 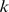. (See Methods - Evaluation Methods for definitions of gene-profile accuracy and other accuracy scores.) As shown, the accuracy is relatively robust for 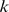 between 10 and 30, while the best accuracy (0.744) is achieved at 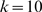. This value of 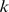 is therefore used in the final model and as a basis for the development of alternative strategy that will be discussed later. Figure 1(b) shows the gene-profile accuracy on randomly chosen genes for each time point. The prediction accuracy is the lowest for the first time point, and increases gradually with time.

Table 1 shows the prediction accuracy on the actual prediction targets for the five top-scoring methods. As can be seen, the method by Gustafsson and Hörnquist (referred to as GH) and KNN have achieved the best prediction accuracy and are far superior to the other methods. The GH method has better gene-profile accuracy than KNN, while KNN has better time-profile accuracy.

**Table 1:** Prediction accuracy on real target genes.

Team	Score	Gene-Profile Accuracy	Gene-Profile P-val	Time-Profile Accuracy	Time-Profile P-val
GH	**3.25**	**0.563**	**6.5E-06**	0.512	4.8E-02
KNN	3.18	0.558	1.1E-05	**0.533**	**3.9E-02**
Team 263	1.85	0.421	7.5E-04	0.112	2.7E-01
Team 297	1.68	0.333	5.6E-03	0.313	7.9E-02
Team 126	1.46	0.324	9.0E-03	0.288	1.4E-01

**Figure 2:**
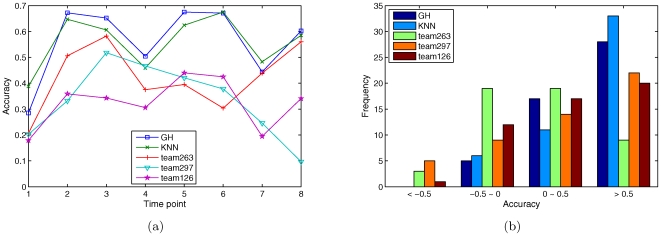
Prediction accuracy on real target genes. (a) Gene-profile accuracy for each time point. (b) Histogram of time-profile accuracy. GH and KNN have almost identical accuracy and are both superior to the other competing methods.

Figure 2(a) shows the gene-profile accuracy on real target genes for each time point. As shown, KNN and GH have better accuracy in nearly all time points than the other methods. On the other hand, the results for all methods are somewhat correlated. Similar to the results on randomly chosen genes, almost all methods have the lowest accuracy at time point 1 (0 minute), indicating the common difficulty for predicting gene expression at this initial time point. Interestingly, the two top-ranked algorithms have achieved very similar results (Pearson correlation = 0.95, 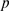-value
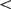
0.0002), even though they are based on very different algorithms and models. Another interesting observation made by comparing Figure 1(b) and Figure 2(a) is that the prediction accuracy on the randomly chosen genes is significantly higher than that on the real target genes. This might be due to the fact that the real target genes are the ones highly perturbed in this experiment, which makes predicting their expression more difficult.

Figure 2(b) shows a histogram of the time-profile accuracy for the 50 target genes. KNN and the GH method are the best again. The overall time-profile accuracy for KNN and GH are 0.53 and 0.51, respectively. These two methods achieved good accuracy (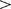
0.5) for 66% and 56% of the genes, respectively, as compared to below 45% for the other methods. The result of KNN is moderately correlated with that of GH (Pearson correlation = 0.52, 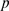-value
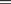
0.0001), and weakly correlated with that of the other three methods (Pearson correlation = 0.18, 0.38, 0.40, 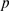-value = 0.22, 0.006, 0.004, respectively).

### Prediction Using Alternative Strategies

Besides the standard KNN algorithm, I also attempted several alternative strategies. All alternative strategies were developed before the gold standard data were released. These results were not submitted to DREAM, however, for various reasons. Two of the alternative strategies did not show better performance than the standard KNN model when tested on randomly selected genes. Another alternative strategy performed better than the standard KNN on randomly selected genes, but the results were obtained a few days after the submission deadline.

The first alternative strategy, called KNN*, is an improved KNN method, where a different number of neighbors may be selected for each gene. This strategy is motivated by the fact that different prediction targets may be involved in different functional pathways, and therefore may be co-expressed with a different number of genes. The idea is similar to a so-called mutual nearest neighbor method [25], where two genes are defined to be neighbors of each other if and only if they are both within the top-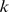 list of the other gene. Here 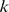 is set to 20, such that in the final co-expression network most prediction targets have around 10 neighbors, a number deemed optimal for the standard KNN model. The actual number of connections for the 50 prediction targets is between 3 and 12, with a mean value of 6.1. Similar prediction results can be obtained using slightly different values of 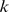 (see below).

The second strategy, referred to as dense subnet, is another KNN-based method. This method first identifies the top-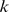 neighbors for each target gene, as in the standard KNN model. Then a subset of these genes densely connected to one another is selected as the true neighbors of the target gene (see Methods). The motivation is that the dense subnetwork around the target gene may represent a functional pathway that the target gene is in; therefore their co-expression to the target gene may be well extrapolated into the prediction strain. On the other hand, genes that are top-ranked but not part of the dense subnetwork may co-express with the target gene only under specific conditions; or the co-expression may be due to noises in the expression data. Therefore, they should not be used to predict the expression of the target gene. For this strategy, 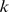 is set to 20, and the final neighborhood size is fixed at 10, so that the results can be compared to that of the standard KNN and KNN* models.

The third strategy is a simple linear regression model, where the expression level of gene 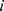 at condition 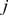 is predicted by the expression levels of the same gene 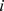 at all other conditions. The same idea is often used for constructing gene regulatory networks, or for imputating missing values in data [15], [21].

The accuracies on randomly chosen genes for the three alternative methods, KNN*, dense subnet, and linear regression, are 0.755, 0.740, and 0.738, respectively, as compared to 0.744 for the standard KNN method. When applied to the actual test data, the overall accuracy is consistent with the accuracy on random genes (Table 2). KNN* significantly improved the accuracy of the standard KNN model, for both gene profiles and time profiles. The dense subnet model has similar gene-profile accuracy as the standard KNN model, but significantly better time-profile accuracy than the latter. The linear regression model has a much worse overall accuracy than the KNN-based methods and the GH method, mainly because of its poor gene-profile accuracy.

**Table 2:** Prediction accuracy of alternative strategies.

Method	Score	Gene-Profile Accuracy	Gene-Profile P-val	Time-Profile Accuracy	Time-Profile P-val
GH	3.25	0.563	6.5E-06	0.512	4.8E-02
KNN	3.18	0.558	1.1E-05	0.533	3.9E-02
KNN*	**3.41**	**0.579**	**5.3E-06**	0.585	**2.8E-02**
Dense subnet	3.27	0.566	9.0E-06	**0.596**	3.2E-02
Linear Regression	2.98	0.542	2.2E-05	0.524	5.1E-02

**Figure 3:**
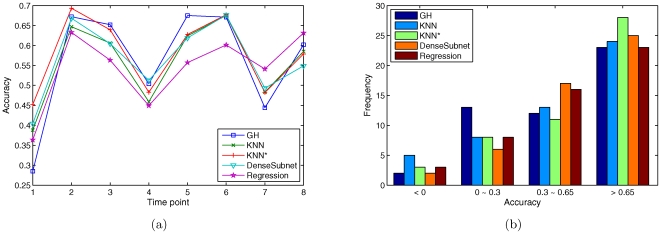
Prediction accuracy of alternative strategies. (a) Gene-profile accuracy for each time point. (b) Histogram of time-profile accuracy.

Figure 3(a) shows the gene-profile accuracy on the real prediction targets at each time point for GH, KNN, and the three alternative strategies. As shown, no algorithm is a clear winner for all time points, and the results of the five algorithms are highly correlated. The Pearson correlation coefficient is above 0.96 between any pair of the three KNN-based methods, and is higher than 0.8 between any pair of the five methods. Figure 3(b) shows the histogram of the time-profile accuracy. As shown, the KNN* method has achieved good accuracy (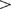
0.65) for the highest percentage of genes (28 out of 50), as compared to 23 out of 50 for GH or linear regression methods. Time-profile accuracies of the three KNN-based methods are also highly correlated (Pearson correlation coefficient = 0.74–0.90, 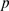-value
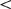
6E-10). Interestingly, time-profile accuracy of the linear regression method is more correlated with that of the standard KNN method (Pearson correlation coefficient = 0.67, 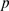-value
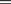
8E-8) than with the GH method (Pearson correlation coefficient = 0.32, 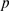-value
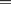
0.02).

The prediction results of all three KNN-based methods are relatively robust with respect to the parameter 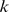. For example, in Table 2, with the default values of 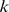, the scores of standard KNN, KNN* and dense subnet are 3.18, 3.41 and 3.20, respectively. In comparison, for 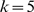 and 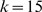, the score of standard KNN is 3.31 and 3.28, respectively. For 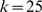 and 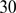, KNN* has a score 3.47 and 3.43, respectively, while dense subnet has a score 3.18 for these two values of 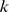.

### Prediction Accuracy Measured by Other Evaluation Methods

As shown in Tables 1 and 2, the gene-profile accuracy is usually higher than the time-profile accuracy. This may be partially due to a problem of the official evaluation method. The prediction results submitted to DREAM were evaluated based on the Spearman correlation between the real and predicted gene expressions. Before calculating correlation, gene expression data were rank-transformed for each time point. However, as these ranks were obtained per time points and therefore may not be directly comparable across time points. As a result, time-profile accuracy may have been under-estimated.

**Figure 4:**
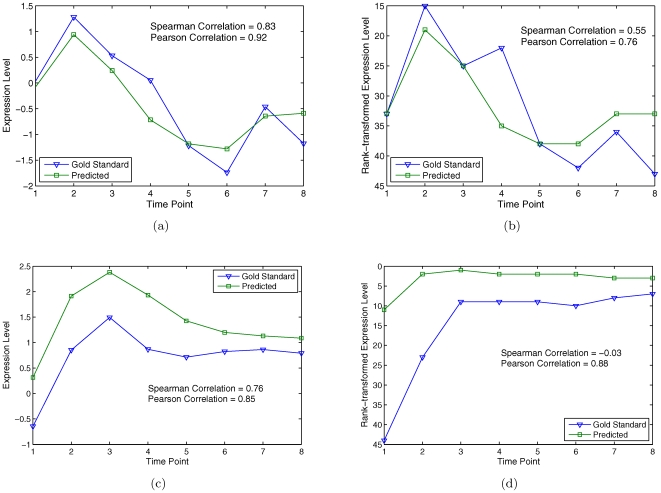
Effects of different scoring methods. (a) Predicted and actual expression levels of YHI9. (b) Predicted and actual rank-transformed expression levels of YHI9. (c) Predicted and actual expression levels of HMX1. (d) Predicted and actual rank-transformed expression levels of HMX1.

To investigate this problem, I obtained the untransformed gene expression data from the data provider, Neil Clarke, and compared the prediction accuracy of the KNN* method on the real prediction targets with four evaluation methods. The first is the official evaluation method used by the DREAM organizers. The second method is also based on rank-transformed gene expression data as in the first method; however it computes Pearson correlation instead of Spearman correlation. (This evaluation method is included only for completeness, as it has the same problem of the official evaluation method.) The third and fourth methods are similar to the first and second methods, respectively, except that gene expression data are not rank-transformed. Note that the first three evaluation methods should result in the same gene-profile accuracy (since ranks are obtained by comparing genes), but potentially different time-profile accuracy. Figure 4 shows two example prediction targets where the actual gene expression levels and the KNN* predicted expression levels are very similar (Figure 4(a) and (c)), while the time-profile accuracy is rather low (Figure 4(b) and (d), Spearman correlation). Table 3 shows the overall scores as well as the time- and gene-profile accuracies of the KNN* method, evaluated by these four methods. Indeed, the official evaluation method resulted in the lowest accuracy (Table 3, rank-transformed expression data and Spearman correlation coefficient). The fourth evaluation method, which uses Pearson correlation coefficient and untransformed data, resulted in the highest accuracy for both time profiles and gene profiles, and the most significant overall scores.

**Table 3:** Accuracy of KNN* using different evaluation methods.

Expression data	Correlation method	Score	Gene-Profile Accuracy	Gene-Profile P-val	Time-Profile Accuracy	Time-Profile P-val
Rank	Spearman	3.41	0.579	5.3E-06	0.585	2.8E-02
Rank	Pearson	3.67	0.579	3.0E-06	0.627	1.5E-02
Value	Spearman	3.59	0.579	3.0E-06	0.610	2.2E-02
Value	Pearson	3.94	0.600	1.6E-06	0.664	7.9E-03

## Discussion

In this article, I presented several simple methods for the DREAM3 gene expression prediction challenge. I treated the challenge problem as a missing value estimation problem rather than a network reverse-engineering problem, and applied existing techniques such as 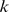-nearest neighbors or linear regression methods to solve it. These simple methods achieved fairly good accuracy, at least when compared with the methods used by the other participants.

The challenge problem seemed daunting at first, especially because the identities of all genes were given explicitly and the DREAM organizers specifically noted that any public data can be utilized. There are overwhelming data available for the yeast genome, including many public microarray data, complete promoter sequences, whole-genome transcription factor binding (ChIP-chip) data, protein-protein interactions, just to name a few. I decided to use only the data provided by the DREAM organizers, because of concerns of inter-data set consistency. It is known that, even though individual high-throughput data set is consistent within itself, consistency between different data sets is usually much lower, especially if they belong to different data types. Therefore, attempting to predict gene expression data from ChIP-chip data, or even to predict gene expression data generated by one lab from expression data generated by a different lab, may turn out to be difficult.

Interestingly, the method by Gustafsson and Hörnquist actually attempted to combine multiple data sources, including ChIP-chip data and public microarray data [22]. By carefully weighting the relative importance of different data sets and using elastic net for soft integration, their method performed slightly better than our simple KNN model. Furthermore, their prediction results are highly correlated with ours. These indicate that the additional data had only marginal contribution towards their predictions.

Several observations made it seemingly desirable to use a gene regulatory network to solve this challenge problem. First, the prediction targets do not seem to be picked randomly. Using a heatmap of the gene expression data, it can be easily seen that the prediction target genes are highly perturbed by 3AT treatment. Second, the three knockout genes are transcription factors, and their binding targets can be obtained from ChIP-chip data. Finally, given the relatively large number of available data points and the small number of target genes, the problem size seems to be reasonable to be handled by the existing network construction algorithms. However, I decided not to pursue gene regulatory networks for this problem, for reasons stated above regarding to inter-data set consistency, and also because most network reconstruction algorithms are model-driven, relying on simplifying model assumptions that are often hard to be tested or fulfilled. For example, methods for constructing regulatory networks must make some simplifying assumptions that may not be true. For example, most methods assume that the mRNA level of a regulator is a true indication of its activity, and that there is no time lag or a constant time lag between the transcription of a regulator and the transcription of its target genes. In reality, some regulators may be regulated post-transcriptionally or on the protein level, with no change on their transcriptional levels. Also, transcriptional time lags between regulators and target genes are not constant and are difficult to estimate in general. In contrast, the simple co-expression-based methods that I have taken assume that gene expression levels in the prediction strain are somewhat correlated with that in the other strains, an assumption that can be easily tested.

It would be very interesting to know what methods the other participants have used, especially the methods that have had inferior performance. Unfortunately, except for the GH method that shared “top performer” status with KNN, details of the other methods are not disclosed, making it hard to speculate why the other methods did not work well. Given that the main theme of the challenge is to evaluate reverse-engineering methods, I suspect some participates have attempted to construct gene regulatory networks. Therefore, the results seem to suggest that at the current stage, although gene regulatory networks are useful at revealing some underlying biology, they can only provide qualitative information. On the other hand, purely data-driven methods, such as the ones used in this work, do not rely on complex model assumptions, and are more useful at making quantitative predictions.

Finally, to benefit the whole scientific community in computational systems biology, I would suggest the organizers of future DREAM competitions to design a mechanism for all participants to provide some key features of their methods (which can be done in an anonymous way). For example, each participant can list the main data types that have been used, and the main idea of the algorithm, or a previous work with similar ideas. It is so important to learn not only from the successes, but also from the failures.

## Methods

### Definitions

Let 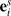 denote the expression vector of the *i*-th probe for strain *s*. The values of ***e*** for all *i* and *s* are given, except for *s* = gat1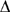, 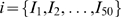. The problem is to predict the missing data, given the available data (as well as any public data).

Define 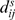 to be the Euclidean distance between the expression vectors of gene *i* and gene *j* in strain *s*: 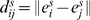.

Also define 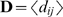 to be a 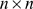 matrix, where 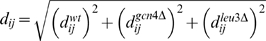 is the distance between the expression vectors of gene 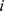 and gene 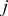 in the three strains having complete data.

### Prediction Models

#### Standard KNN model

Let 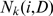 be the 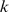 nearest neighbors of gene 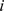, defined based on the distance matrix 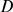. Note that, 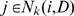 does not imply that 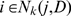. Prediction targets are prohibited from being selected as neighbors of other prediction targets.

In the standard KNN model, the expression level of gene 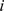 at time point 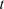 is estimated by the average expression level of its 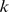 nearest neighbors:
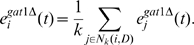



#### Improved KNN model

In the standard KNN model described above, the expression level of a gene is predicted by the average of 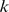 genes. However, in reality, different genes may be involved in different functional pathways which may have different sizes. Therefore, I propose an improved KNN model, called KNN*, which may select a different number of neighbors for each gene.

Let 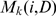 be a subset of the 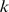 nearest neighbors of gene 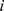 such that for any 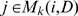 I have 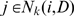 and 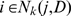. The graph defined by 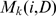 is known as mutual nearest neighbor graph [25]. Compared to the standard 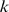-nearest neighbor graph, the mutual KNN requires an edge to be confirmed by both nodes involved in the edge. As a result, each node may end up with a different number of edges. This model may be more realistic compared to the standard KNN model as the former does not assume each gene to have the same number of co-expressed genes. However, in the mutual KNN model, some prediction targets may have no neighbors at all, which is undesirable. Therefore, I require that each prediction target be connected to its top three nearest neighbors as in the standard KNN graph, regardless of their appearance in the mutual KNN graph. In this model, the neighborhood of gene 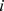 is defined as 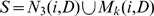. The expression level of gene 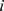 at time point 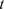 can be estimated accordingly:
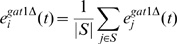



#### Dense subnet model

In this model, the neighbors are identified heuristically as follows. I first construct a standard KNN network using all genes on the chip, with 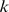 fixed at 20. For each target gene 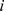, a subnetwork containing its top-20 nearest neighbor genes is obtained from the global KNN network. I then rank the 20 genes according to their connectivity within this sub-network, and select the 10 nodes with the highest connectivity. Since these 10 genes are all connected to the target gene 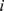, and have relatively more interactions within the group, they form a dense subnetwork around gene 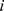. Defining this set of genes as the true neighbors of the target gene, the expression of the target gene is then predicted by the same formula as in the standard KNN model.

#### Linear regression model

The linear regression model assumes that the expression level of a prediction target in the gat1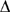 strain is related to its own expression levels in the other three strains, and can be estimated by a linear combination of the 3×8 = 24 gene profiles from the three strains:
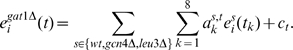
For each time point 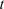, the constants 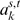 and 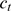 are estimated by solving a linear regression using the genes without missing data.

### Evaluation Methods

#### Evaluation method proposed by DREAM organizers

The prediction results submitted to DREAM are evaluated based on the Spearman correlation coefficient between the real and predicted gene expression profiles. Before calculating the correlation coefficient, the gene expression data are rank-transformed for each time point. Specifically, for any given time point, the prediction target with the highest induction has a rank 1 and the gene with the highest repression has a rank 50. Given the rank-transformed gene expression, a **gene profile** is defined as the ranks for all genes to be predicted at a given time point, and a **time profile** is defined as the ranks for a single gene across all time points. The **gene-profile accuracy** for a given time point is defined as the Spearman correlation coefficient between the actual gene profile and the predicted gene profile. Correspondingly, the **gene-profile** 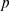 **-value** for a given time point is the probability that a given or larger Spearman correlation coefficient can be achieved by randomly ordered ranks. The **overall gene-profile accuracy** is defined as the average gene-profile accuracy across all eight time points, while the **overall gene-profile** 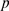 **-value** is defined as the geometric mean of the individual gene-profile 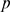-values. The **time-profile accuracy** and **time-profile** 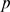 **-value** are defined similarly, except that the measurement is based on time profiles instead of gene profiles. The **score** of a prediction algorithm is computed as −0.5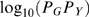, where 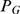 and 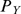 are the overall gene-profile 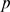-value and the overall time-profile 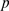-value, respectively. A larger score indicates greater statistical significance of the prediction.

#### Alternative evaluation methods

In the evaluation method proposed by the DREAM organizers, Spearman correlation coefficient is computed for rank-transformed expression data. However, since the ranks are obtained for each time point, they are not comparable across time points. Therefore, the estimated time-profile accuracy may be inaccurate. To address this problem, I propose two additional scoring methods, using the raw gene expression data rather than the rank-transformed expression data. The first additional method computes Spearman correlation coefficient between the predicted and actual raw (instead of rank-transformed) gene expression values. The second method also relies on the raw expression data, but computes Pearson correlation coefficient rather than Spearman correlation coefficient. For completeness, I also propose a method that computes Pearson correlation coefficient between rank-transformed expression values, which has the same pitfall as the original evaluation methods. The impact of these different evaluation methods on the prediction accuracy of real target genes is shown in the Results section (Table 3 and Figure 4).

### Model Selection

In the model development stage, I use randomly chosen genes to estimate the prediction accuracy of different models and to select the optimal model parameters. For each model, I first randomly pick 50 genes that are not the prediction targets, and remove their expression data in gat1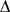. I then predict their expression levels using the model, and computed the overall gene-profile accuracy. This process is repeated 10 times for each model, and the average accuracy is used to evaluate and select models. Only gene-profile accuracy is considered at model selection stage, as the time-profile accuracy depends on the set of genes selected, and therefore may not be a good indicator for the accuracy of the real prediction targets, which may have been selected because of their special roles under these experimental conditions.
